# Time-dependent changes in aerobic capacity of acute sub-high altitude exposure in elite youth endurance athletes

**DOI:** 10.3389/fphys.2026.1916986

**Published:** 2026-08-19

**Authors:** Junpeng Feng, Fengming Ning, Xuchang Zhang, Ye Tao, Mingyue Sun, Wenqiang Wu

**Affiliations:** 1 China Winter Sports College, Beijing Sport University, Beijing, China; 2 College of Education, Beijing Sport University, Beijing, China; 3 Department of Physical Education, Beijing City University, Beijing, China

**Keywords:** endurance, hypoxic acclimatization, running economy, sub-high altitude, youth athletes

## Abstract

**Purpose:**

This study investigated the non-linear, time-dependent alterations in cardiorespiratory fitness, running economy (RE), and metabolic kinetics during the first week of acute exposure to a sub-high altitude (1,300 m) in elite youth endurance athletes.

**Methods:**

Fourteen male national-level middle- and long-distance runners (age: 19.8 ± 0.7 years; sea-level $\dot{V}{\text{O}}_{2\text{max}}$: 67.2 ± 3.8 mL·kg^-^¹·min^-^¹) completed field-based incremental exhaustion tests on a standard 400-m outdoor track at sea level (T0) and on day 1 (T1, within 18 h post-arrival), day 4 (T2), and day 6 (T3) after rapid rail-based ascent to 1,300 m. Maximal oxygen uptake ( $\dot{V}{\text{O}}_{2\text{max}}$), peak running speed ( $v\dot{V}{\text{O}}_{2\text{max}}$), and submaximal RE (steady-state $\dot{V}{\text{O}}_{2}$ at 12, 14, and 16 km·h^-^¹) were assessed using an automated metabolic cart. Blood lactate concentration ([La^-^]b) and maximal heart rate (HRmax) were measured. Daily waking morning heart rate (morning HR) and resting arterial oxygen saturation (SpO_2_) were continuously monitored. Training volume was strictly standardized to 70% of the habitual sea-level load.

**Results:**

One-way RM ANOVA revealed a significant main effect of time on $\dot{V}{\text{O}}_{2\text{max}}$ ( $F[3,39]=18.42,p<0.001,\eta_{p}^{2}=0.59$). Crucially, polynomial contrasts confirmed a highly significant quadratic trend ( $F_{\text{quadratic}}[1,13]=34.21,p_{\text{trend}}<0.001,\eta_{p}^{2}=0.72$), statistically validating a non-linear V-shaped temporal kinetic profile. $\dot{V}{\text{O}}_{2\text{max}}$ dropped from ${\text{T}}_{1}$ to a distinct nadir at ${\text{T}}_{2}$ ( −7.0%, $95\%\text{ CI}:[60.1,64.7]\;\text{mL}\cdot {\text{kg}}^{-1}\cdot {\text{min}}^{-1},p<0.001$), and partially rebounded at ${\text{T}}_{3}$. Concurrently, submaximal RE at 14 km/h significantly deteriorated at ${\text{T}}_{2}$, as evidenced by a 5.3% increase in distance-specific oxygen cost (from 210.5±12.4 at ${\text{T}}_{1}$ to $221.7\pm 14.2\;\text{mL}\cdot {\text{kg}}^{-1}\cdot {\text{km}}^{-1}$ at ${\text{T}}_{2}$, p=0.010), which also exhibited a significant quadratic pattern ( $p_{\text{trend}}=0.008$). Post-exercise blood lactate peaked at ${\text{T}}_{2}$ ( $11.2\pm 1.4\;\text{mmol}\cdot {\text{L}}^{-1},p=0.015$), while ${\text{HR}}_{\text{max}}$ remained statistically stable ( $F[3,39]=0.21,p=0.885,\eta_{p}^{2}=0.015$).

**Conclusion:**

Acute relocation to 1,300 m induces a distinct physiological nadir on Day 4, which may be related to transient autonomic adjustments and respiratory muscle work, rather than hematological adaptations. Early adaptation emerges by Day 6. Coaches may consider closer monitoring and temporary adjustment of training intensity during the early days following ascent, particularly around day 4.

## 1 Introduction

Altitude training paradigms have been extensively used by elite endurance athletes for decades to optimize both hypoxic and sea-level aerobic power through a combination of hematological and non-hematological adaptations (Levine and Stray-Gundersen, 1997; Millet et al., 2010). However, sudden exposure to low-oxygen environments induces immediate, severe homeostatic stress. This stress is primarily driven by a drop in barometric pressure ( $P_{B}$), which reduces the inspired partial pressure of oxygen ( $P_{I}{\text{O}}_{2}$) and restricts the entire convective oxygen transport cascade from alveolar diffusion to mitochondrial oxidative phosphorylation (Hochachka and Somero, 2002; West, 2012; Calbet et al., 2015). While physiological adjustments at high altitudes ( >2,500 m) and moderate altitudes ( 1,500– 2,500 m) have been extensively mapped (Wehrlin and Hallén, 2006; Gore et al., 2013), the systemic and metabolic time-course responses to sub-high altitudes ( 1,000– 1,500 m) remain poorly understood and are frequently neglected in mainstream sports medicine.

Sub-high altitude regions are of immense practical importance because many international elite training centers and high-level competition venues are located within this specific geographic band, including Kunming (China), Nairobi (Kenya), Addis Ababa (Ethiopia), and several European locations (Chapman et al., 2014; Garvican-Lewis et al., 2015). Although sub-high altitude elevations do not provoke severe clinical pathologies such as acute mountain sickness (AMS), accumulating evidence confirms that they are sufficient to alter gas exchange kinetics, arterial oxygen saturation ( ${\text{SpO}}_{2}$), and running economy (RE) in highly trained athletes (Saunders et al., 2004; Clark et al., 2004, 2007). In elite endurance sports, where performance differentials are decided by fractions of a percent, even a minor impairment in aerobic capacity can dictate competitive outcomes (Hopkins et al., 1999). Therefore, identifying the exact temporal kinetics of performance degradation and early stabilization during the first week post-ascent within this elevation zone is a critical empirical requirement (Bonetti and Hopkins, 2009).

Previous research at moderate-to-high elevations suggests that early hypoxic adaptation is non-linear. These studies show a significant drop in maximal oxygen uptake ( $\dot{V}{\text{O}}_{2\text{max}}$) and movement efficiency, typically peaking between days 3 and 5 post-ascent, driven by acute ventilatory, autonomic, and hemorheological shifts (Robach et al., 2006; Schuler et al., 2007). Following this transient deficit, early physiological stabilization begins, mediated by ventilatory acclimatization and renal bicarbonate clearance (Bärtsch and Saltin, 2008; Siebenmann et al., 2012). However, this timeline exhibits considerable individual variance based on age and athletic maturity (Stray-Gundersen et al., 2001; Friedmann et al., 2005). Notably, elite youth distance runners represent a highly specialized cohort characterized by ongoing cardiorespiratory development, distinct autonomic nervous system reactivity, and unique metabolic profiles, including lower initial lactate production capacities and faster recovery rates compared to mature adults (Armstrong and Barker, 2011; Ratel et al., 2015; Slessarev et al., 2010). To date, longitudinal research detailing the daily physiological and metabolic tracking of elite youth endurance athletes during acute sub-high altitude exposure remains non-existent.

Furthermore, the existing altitude training literature is constrained by low-frequency testing intervals, typically comparing pre-ascent values with a single time point after 1 or 2 weeks of exposure (Fudge et al., 2007; Saugy et al., 2014). This sparse sampling misses the critical micro-regulatory adjustments occurring within the first 7 days, leaving a major blind spot for coaches who must design optimal training intensities immediately upon arrival (Chapman et al., 2016). Acute low-oxygen exposure triggers immediate hyperventilation via peripheral chemoreceptors, which elevates resting cardiac work and increases respiratory muscle energy costs, temporarily compromising systemic movement efficiency (running economy, RE) (Gore et al., 2007; Saunders et al., 2009). Understanding how these complex interactions between central cardio-ventilatory work and peripheral muscle metabolism manifest over consecutive days at 1,300 m is essential for optimizing acute athletic preparation.

Therefore, this study aimed to determine the precise time-dependent alterations in cardiorespiratory fitness, running economy, metabolic responses, and resting autonomic/oxygenation markers in elite youth distance runners during the first week following rapid relocation to a sub-high altitude ( 1,300 m). Based on the principles of acute low-oxygen physiology, we hypothesized that these athletes would experience a transient but substantial performance nadir around day 4, characterized by concurrent depressions in peripheral oxygenation and an increased reliance on anaerobic glycolysis, followed by early homeostatic re-stabilization by day 6.

## 2 Materials and methods

### 2.1 Participants

Fourteen elite male youth middle- and long-distance runners volunteered to participate in this study. All participants were registered national-level athletes with a minimum of 4 years of structured endurance training history. Their mean baseline characteristics were: age 19.8±0.7 years; body mass 62.3±4.1 kg; height 176.4±5.2 cm; and sea-level $\dot{V}{\text{O}}_{2\text{max}}$ $67.2\pm 3.8\text{ mL}\cdot {\text{kg}}^{-1}\cdot {\text{min}}^{-1}$. Exclusion criteria included: (1) any history of altitude exposure above 500 m within the preceding 3 months; (2) musculoskeletal injuries or illnesses during the experimental timeline; and (3) use of medications or supplements known to alter cardiorespiratory or metabolic functions. The study protocol was approved by the Institutional Human Research Ethics Committee, and written informed consent was obtained from all athletes and their legal guardians prior to data collection.

### 2.2 Experimental design and altitude relocation protocol

The study employed a longitudinal, single-cohort design. Baseline sea-level controls (T0) were established at an elevation of <100 m. Following baseline testing, the cohort was rapidly transported via high-speed rail to a sub-high altitude training venue located at 1,300 m within 6 h. Laboratory-controlled exercise testing sessions were performed on day 1 (T1, within 18 h post-arrival), day 4 (T2), and day 6 (T3). To isolate the effects of environmental hypoxia, dietary intake, sleep duration, and training loads were strictly standardized. Total training volume during the 6-day exposure was clamped at 70% of the athletes’ habitual sea-level training volume, consisting exclusively of low-to-moderate intensity regenerative running.

### 2.3 Daily acclimatization monitoring

Every morning immediately upon waking, participants underwent resting physiological assessments in a supine position. Waking morning heart rate (morning HR) was recorded using a chest-strap monitor. Resting peripheral arterial oxygen saturation ( ${\text{SpO}}_{2}$) was determined with a medical-grade pulse oximeter placed on the index finger.

### 2.4 Cardiorespiratory fitness and metabolic assessment

All exercise tests were conducted on a standard 400-m outdoor track under stable environmental conditions. Participants performed a 15-min light jogging warm-up at a self-selected comfortable pace, followed by a 20-min rest period to allow physiological parameters to return to near-baseline levels. A portable breath-by-breath gas exchange system (Cortex MetaMax 3B R2, Germany) was used to continuously measure oxygen uptake (V̇O_2_), carbon dioxide production (V̇CO_2_), and minute ventilation (V̇E). The device was calibrated before each test using ambient air and a standard gas mixture according to the manufacturer’s instructions. All tests were performed at the same time of day for each athlete to minimize circadian variation.

Incremental exercise protocol: The test consisted of continuous running with stepwise speed increments as follows:

- One lap (400 m) at 6 km·h^-^¹
- One lap (400 m) at 8 km·h^-^¹
- Two laps (800 m) at 10 km·h^-^¹
- Thereafter, speed was increased by 2 km·h^-^¹ every two laps (800 m) until volitional exhaustion.

Speed control and pacing: During the field-based incremental exhaustion tests, a calibrated bicycle equipped with a power meter was used to guide running speed throughout the entire test. The cyclist maintained a constant speed corresponding to the target running pace for each stage. Additionally, test personnel provided auditory pacing reminders every 200 m, informing the athlete of the deviation from the target pace.

Maximal oxygen uptake ( $\dot{V}{\text{O}}_{2\text{max}}$): $\dot{V}{\text{O}}_{2\text{max}}$ was recorded as the highest 30-s average $\dot{V}{\text{O}}_{2}$ (mL·kg^-^¹·min^-^¹) attained during the test. Achievement of $\dot{V}{\text{O}}_{2\text{max}}$ was confirmed when at least two of the following criteria were met: (1) a plateau in $\dot{V}{\text{O}}_{2}$ (increase <2.0 mL·kg^-^¹·min^-^¹) despite a further increase in speed; (2) a peak respiratory exchange ratio (RER) ≥1.10; (3) heart rate within 10 bpm of the age-predicted maximum (220 − age); (4) a rating of perceived exertion (RPE, Borg 6–20 scale) ≥18.

Peak running speed (vV̇O_2_max): vV̇O_2_max (km·h^-^¹) was defined as the highest running speed that the participant sustained for at least one full lap (400 m) immediately before volitional exhaustion.

Maximal heart rate (HRmax): HRmax (bpm) was the highest heart value recorded during the incremental test, measured using the heart rate monitor integrated into the Cortex MetaMax 3B R2 system.

Running economy (RE): Running economy was assessed at standardized submaximal speeds of 12, 14, and 16 km·h^-^¹. At each speed, oxygen uptake was averaged over the final 60 s of the stage to ensure that a physiological steady state was achieved. RE was expressed as the steady-state oxygen cost of running in mL·kg^-^¹·min^-^¹, averaged over the two laps completed at each submaximal speed, using data from the portable gas analysis system.

Blood lactate concentration (BLa): Capillary blood samples were collected from the earlobe at the following time points: (1) at rest, 5 min before the warm-up; (2) immediately after completion of each submaximal running stage (i.e., at the end of each 2-lap segment at 12, 14, and 16 km·h^-^¹); and (3) immediately (within 1 min) after the completion of the incremental exhaustion test. At each sampling point, the athlete momentarily stopped running for capillary blood collection; the interval between stage completion and blood collection was standardized to <30 s. The sampling procedure was identical at all time points. Blood lactate concentration (mmol·L^-^¹) was analyzed using a portable lactate analyzer (EKF Scout4, Germany), which was calibrated daily according to the manufacturer’s instructions.

### 2.5 Statistical analysis

Data are reported as mean ± standard deviation (SD). Data normality and sphericity assumptions were verified using the Shapiro-Wilk and Mauchly’s tests, respectively. When the sphericity assumption was violated, the Greenhouse-Geisser correction was applied. To statistically validate the hypothesized non-linear “decline-nadir-rebound” temporal kinetic arc, polynomial contrasts (linear and quadratic) were embedded within the one-way repeated-measures ANOVA framework. *Post-hoc* pairwise comparisons were executed using the Bonferroni correction. Effect sizes were quantified using partial eta-squared (ηp²), and 95% confidence intervals (95% CI) were reported for all primary outcomes to enhance statistical transparency. Statistical significance was defined as α = 0.05. All analyses were performed using SPSS 26.0 (IBM Corp., Armonk, NY, USA).

## 3 Results

The physiological, efficiency, metabolic, and waking homeostatic variables exhibited highly distinct, non-linear time-dependent alterations during the first week of acute sub-high altitude exposure.

### 3.1 Cardiorespiratory fitness and maximal aerobic power dynamics

Cardiorespiratory fitness and maximal aerobic power dynamics across time points are presented in **Table 1**. Acute exposure to 1,300 m triggered a highly synchronized, non-linear kinetic wave in cardiorespiratory capacity. One-way RM ANOVA demonstrated a significant main effect of time on $\dot{V}{\text{O}}_{2\text{max}}$ ( $F[3,39]=18.42,p<0.001,\eta_{p}^{2}=0.59$). Importantly, polynomial trend analysis verified a highly significant quadratic component ( $F_{\text{quadratic}}[1,13]=34.21,p_{\text{trend}}<0.001,\eta_{p}^{2}=0.72$), indicating curvature across the selected measurement points. > During the standard outdoor track field-based incremental exhaustion tests, $\dot{V}{\text{O}}_{2\text{max}}$ at ${\text{T}}_{1}$ ( $67.1\pm 3.7\;\text{mL}\cdot {\text{kg}}^{-1}\cdot {\text{min}}^{-1}$, 95% CI:[64.8,69.4]) was statistically indistinguishable from the baseline. However, cumulative hypoxic stress provoked a system-wide nadir at ${\text{T}}_{2}$ ( $62.4\pm 3.9\;\text{mL}\cdot {\text{kg}}^{-1}\cdot {\text{min}}^{-1}$, 95% CI:[60.1,64.7]), exhibiting a 7.0% deficit ( p<0.001). By ${\text{T}}_{3}$ (Day 6), a robust homeostatic rebound expanded $\dot{V}{\text{O}}_{2\text{max}}$ back to $65.0\pm 3.5\;\text{mL}\cdot {\text{kg}}^{-1}\cdot {\text{min}}^{-1}$ ( 95% CI:[62.9,67.1]), which significantly transcended the ${\text{T}}_{2}$ nadir ( p=0.042) and closed the deficit gap with baseline to a non-significant 3.1% ( p=0.070).

**Table 1:** Cardiorespiratory fitness and maximal aerobic power dynamics across time points.

Time point	$\dot{V}{\text{O}}_{2\text{max}}$ (mL·kg^-^¹·min^-^¹)	$\dot{V}{\text{O}}_{2\text{max}}$ % change vs. T0	$v\dot{V}{\text{O}}_{2\text{max}}$ (km·h^-^¹)
T0 (sea level baseline)	67.2 ± 3.8	Baseline	20.0 ± 0.5
T1 (altitude day 1)	67.1 ± 3.7	–0.1%	19.8 ± 0.4
T2 (altitude day 4)	$62.4\pm 3.9^{**}$	–7.0%	$18.5\pm 0.6^{*}$
T3 (altitude day 6)	$65.0\pm 3.5^{\#}$	–3.1%	19.2±0.5
F/ p	F=18.42, p<0.001	–	F=6.12, p<0.05

*Data are mean ± SD ( n=14). * p<0.05 vs. T0; * p<0.01 vs. T0; # p=0.07 (adaptive recovery trend relative to T2).

Peak running speed (vV̇O_2_max) mirrored this quadratic trajectory (F[3,39] = 6.12, p < 0.05, ηp² = 0.53; ptrend < 0.001, ηp² = 0.63), bottoming out at T2 (18.5 ± 0.6 km·h^-^¹, 95% CI: [17.8,18.6]) and significantly reviving by T3 (19.2 ± 0.5 km·h^-^¹). .

### 3.2 Running economy and submaximal efficiency profiles

Submaximal running economy data at fixed velocities are summarized in **Table 2**. Steady-state oxygen cost at fixed submaximal speeds was significantly altered by time ( p=0.01). At T2, running economy (RE) worsened markedly, as shown by a 5.3% increase in oxygen consumption at $14\text{ km}\cdot {\text{h}}^{-1}$ compared with T1 ( 51.7±2.8 vs. $49.1\pm 2.5\text{ mL}\cdot {\text{kg}}^{-1}\cdot {\text{min}}^{-1}$, p=0.01). By day 6 (T3), submaximal oxygen requirements fell back to $49.8\pm 2.3\text{ mL}\cdot {\text{kg}}^{-1}\cdot {\text{min}}^{-1}$, demonstrating rapid re-stabilization of running efficiency ( p>0.05 vs. T0).

**Table 2:** Submaximal running economy ( $\dot{V}{\text{O}}_{2}$) at fixed velocities.

Time point	$\dot{V}{\text{O}}_{2}$ at 12 km·h^-^¹ (mL·kg^-^¹·min^-^¹)	$\dot{V}{\text{O}}_{2}$ at 14 km·h^-^¹ (mL·kg^-^¹·min^-^¹)	$\dot{V}{\text{O}}_{2}$ at 16 km·h^-^¹ (mL·kg^-^¹·min^-^¹)
T0 (sea level baseline)	41.8 ± 1.9	49.0 ± 2.4	56.5 ± 2.8
T1 (altitude day 1)	42.0 ± 2.1	49.1 ± 2.5	56.9 ± 3.0
T2 (altitude day 4)	$43.9\pm 2.4^{*}$	$51.7\pm 2.8^{*}$	$59.4\pm 3.2^{*}$
T3 (altitude day 6)	42.2 ± 1.8	49.8 ± 2.3	57.2 ± 2.7
p-value	<0.05	=0.01	<0.05

*Data are mean ± SD ( n=14). p<0.05 *vs. T0.*

Concurrently, post-exercise blood lactate (BLa) demonstrated an identical inverted V-shaped quadratic burst ( $F[3,39]=5.43,p=0.015,\eta_{p}^{2}=0.29$; $p_{\text{trend}}=0.011$), climbing to a peak of $11.2\pm 1.4\;\text{mmol}\cdot {\text{L}}^{-1}$ ( 95% CI:[10.4,12.0]) at ${\text{T}}_{2}$, confirming a compensatory upregulation of anaerobic glycolytic flux during the aerobic nadir window (Brooks et al., 1991).

### 3.3 Metabolic and chronotropic responses during incremental testing

Metabolic lactate accumulation and maximal heart rate characteristics are shown in **Table 3**. Blood lactate responses indicated an increased reliance on anaerobic energy pathways during submaximal running. At a fixed speed of $16\text{ km}\cdot {\text{h}}^{-1}$, ${\left[{\text{La}}^{-}\right]}_{\text{b}}$ rose sharply from $4.2\pm 0.7\text{ mmol}\cdot {\text{L}}^{-1}$ at T0 to a peak of $5.8\pm 1.0\text{ mmol}\cdot {\text{L}}^{-1}$ at T2 ( p=0.02), before receding to $4.7\pm 0.9\text{ mmol}\cdot {\text{L}}^{-1}$ at T3. Post-exhaustion maximal blood lactate followed an identical kinetic arc, peaking significantly on day 4 (11.2 ± 1.4 mmol·L^-^¹, p = 0.015). Conversely, maximal chronotropic capacity was unaffected by sub-high altitude exposure, with ${\text{HR}}_{\text{max}}$ remaining statistically identical across all testing intervals ( p>0.05).

**Table 3:** Metabolic lactate accumulation and maximal heart rate characteristics.

Time point	Submaximal ${\left[{\text{La}}^{-}\right]}_{\text{b}}$ at 16 km·h^-^¹ (mmol·L^-^¹)	Maximal post-exhaustion ${\left[{\text{La}}^{-}\right]}_{\text{b}}$ (mmol·L^-^¹)	${\text{HR}}_{\text{max}}$ (bpm)
T0 (sea level baseline)	4.2 ± 0.7	9.5 ± 1.1	198 ± 5
T1 (altitude day 1)	4.4 ± 0.8	9.7 ± 1.2	198 ± 6
T2 (altitude day 4)	$5.8\pm 1.0^{*}$	$11.2\pm 1.4^{*}$	197 ± 5
T3 (altitude day 6)	4.7 ± 0.9	10.1 ± 1.1	198 ± 5
p-value	=0.02	=0.15	>0.05

*Data are mean ± SD ( n=14). p<0.05 *vs. T0.*

### 3.4 Resting acclimatization indices and autonomic markers

Daily waking morning heart rate and arterial oxygen saturation kinetics are detailed in **Table 4**. Daily resting physiological parameters confirmed a significant homeostatic disruption at T2. Waking ${\text{SpO}}_{2}$ dropped from a sea-level baseline of 98.6±0.4% to its lowest point on day 4 ( 94.1±1.2%, p<0.001), followed by an adaptive upward trend on day 6 ( 96.2±0.8%). Mirroring the oxygenation changes, morning heart rate (HR) elevated significantly at T2, rising by approximately 17.6% above sea level ( 60±6 bpm vs. 51±4 bpm, p<0.01), before resolving toward baseline levels at T3 ( 55±4 bpm).

**Table 4:** Daily waking morning heart rate and arterial oxygen saturation kinetics.

Time point	Waking ${\text{SpO}}_{2}$ (%)	Waking morning HR (bpm)
T0 (sea level baseline)	98.6 ± 0.4	51 ± 4
T1 (altitude day 1)	95.5 ± 0.9	$57\pm 5^{*}$
T2 (altitude day 4)	$94.1\pm 1.2^{†}$	$60\pm 6^{**}$
T3 (altitude day 6)	96.2 ± 0.8	$55\pm 4^{\#}$
p-value	<0.001	<0.01

*Data are mean ± SD ( n=14). * p<0.05 vs. T0; * p<0.01 vs. T0; † p<0.05 vs. T1; #Adaptive recovery trend relative to T2.

## 4 Discussion

The primary finding of this investigation is that acute exposure to a sub-high altitude environment (1,300 m) induces a highly specific, non-linear pattern of physiological strain in elite youth endurance runners. This pattern is characterized by a significant performance degradation that peaks on day 4 (T2) before undergoing early homeostatic restoration by day 6 (T3). This study is the first to demonstrate that even a modest elevation gain (1,300 m) is sufficient to elicit a pronounced physiological nadir in youth athletes. This timeline reflects the structural trends previously reported at much higher altitudes but operates within a narrow homeostatic regulatory band (Wehrlin and Hallén, 2006; Clark et al., 2007).

### 4.1 Mechanistic basis of the day 4 aerobic nadir

The 7.0% reduction in $\dot{V}{\text{O}}_{2\text{max}}$ observed on day 4 represents a profound systemic limitation in the convective oxygen transport cascade. Upon rapid ascent to a low-oxygen environment, the immediate reduction in $P_{I}{\text{O}}_{2}$ lowers alveolar oxygen tension ( $P_{A}{\text{O}}_{2}$), narrowing the diffusion gradient across the alveolar-capillary membrane (Beall, 2007; Calbet et al., 2015). Daily waking physiological tracking supported this mechanism, demonstrating that resting arterial oxygen saturation ( ${\text{SpO}}_{2}$) reached its absolute lowest point (94.1%) on day 4. This hypoxemic state reduces arterial oxygen content ( $C_{a}{\text{O}}_{2}$), restricting the maximum volume of oxygen delivered to the working locomotor muscles (Ferguson et al., 2018; Robach et al., 2006; Siebenmann et al., 2012).

Crucially, $\dot{V}{\text{O}}_{2\text{max}}$ did not drop significantly on day 1 (T1). This temporary preservation of maximal aerobic power may be attributed to acute neuro-endocrine compensations. Immediate hypoxic exposure triggers a wave of sympathetic nervous system excitation, driving a surge in circulating catecholamines (norepinephrine and epinephrine) (Mazzeo et al., 1998; Hansen and Sander, 2003). This systemic neural drive maintains cardiac output ( $\dot{Q}$) during exercise by elevating submaximal heart rate, temporarily sustaining muscle oxygen flux despite a reduced $C_{a}{\text{O}}_{2}$ (Stray-Gundersen et al., 2001). However, by day 4 (T2), this initial sympathetic drive may undergo receptor down-regulation—a well-known phenomenon termed sympathetic withdrawal or desensitization—while long-term structural adaptations, such as erythropoietin (EPO)-mediated red blood cell mass expansion, have not yet developed (Ekblom et al., 1975; Lundby et al., 2012; Richardson et al., 2020). This temporal gap between neural habituation and hematological adaptation could contribute to the severe physiological deficit observed on day 4 (Green et al., 1992).

### 4.2 Biomechanical and metabolic efficiency adjustments

The transient physiological collapse observed on Day 4 (T2) is hypothesized to be driven by short-term acute regulatory and autonomic pathways. Instead, this transient physiological collapse is hypothesized to be driven by short-term acute regulatory and autonomic pathways. First, acute exposure to sub-high altitude accelerates the hypoxic ventilatory response (HVR). In our field-based tests conducted on an outdoor running track, this severely increased hyperpneic breathing work. This massive mechanical demand of ventilation may trigger the respiratory muscle metaboreflex (Amann et al., 2006; Harms et al., 1997). This reflex potentially upregulates sympathetic vasoconstrictor outflow, shunting oxygenated blood away from the active locomotor skeletal muscles toward the highly loaded diaphragm and intercostal muscles (Babcock et al., 1995). This temporary “perfusion conflict” between respiratory and locomotor muscles could explain the concurrent 5.3% reduction in running economy and the compensatory rise in blood lactate ( $11.2\pm 1.4\;\text{mmol}\cdot {\text{L}}^{-1}$) observed at ${\text{T}}_{2}$. Second, the rapid homeostatic recovery observed by Day 6 ( ${\text{T}}_{3}$) highlights the unique developmental physiology and superior autonomic plasticity characteristic of elite youth athletes. Compared to older adult counterparts, adolescents typically possess a highly resilient parasympathetic reactivation capacity and rapid metabolic/mitochondrial remodeling potentials. This may allow them to quickly restore acid-base balance (renal ${\text{HCO}}_{3}^{-}$ compensation), reset respiratory sensitivity, and downregulate hypersympathetic drive within 48 hours following the peak cardiovascular stress window.

### 4.3 Integrity of myocardial chronotropic capacity

A notable finding in this cohort is the absolute stability of maximal heart rate ( ${\text{HR}}_{\text{max}}$) across all testing intervals (197–198 bpm, p>0.05). At high altitudes ( >3,000 m), ${\text{HR}}_{\text{max}}$ consistently falls due to increased parasympathetic drive or myocardial β-adrenergic receptor desensitization—an evolutionary adaptation thought to protect cardiac tissue from severe ischemic stress (Boushel et al., 2001; Richalet et al., 2012). The complete lack of alteration in ${\text{HR}}_{\text{max}}$ at 1,300 m indicates that sub-high altitude exposure does not impair central myocardial chronotropic power. Therefore, the observed declines in $\dot{V}{\text{O}}_{2\text{max}}$ may be related to peripheral oxygen extraction deficits and convective oxygen limitations rather than by central cardiac pump constraints (Wehrlin and Hallén, 2006; Calbet et al., 2015).

### 4.4 Developmental and practical training frameworks

From a developmental perspective, elite youth runners exhibit unique adaptation profiles compared with historical adult data. Youth athletes possess higher baseline autonomic plasticity and faster systemic recovery kinetics, driven by rapid capillary and mitochondrial remodeling capacities (Armstrong and Barker, 2011; Ratel et al., 2015). This youthful plasticity explains the rapid homeostatic stabilization observed by day 6 (T3), where submaximal RE returned to baseline and $\dot{V}{\text{O}}_{2\text{max}}$ recovered to within 3.1% of sea-level controls. This early recovery is supported by rapid renal bicarbonate excretion ( ${\text{HCO}}_{3}^{-}$), which normalizes blood pH and restores respiratory efficiency (Siebenmann et al., 2012; Chapman et al., 2014).

Mapping this daily adaptation timeline provides clear periodization guidelines for coaches managing youth distance runners at sub-high altitudes. The presence of a physiological nadir on day 4 confirms that scheduling high-intensity anaerobic training, maximal lactate accumulation trials, or major selection events during this period will cause excessive fatigue and elevate injury risk (Chapman et al., 2016; Wilber, 2007). Conversely, days 1–2 represent a usable initial training window supported by acute sympathetic drive, while day 4 must be strictly reserved for active recovery and regenerative sessions. High-intensity training volume can safely resume on day 6, aligning with early homeostatic re-stabilization.

### 4.5 Interindividual variation

The observation that group-average physiological values were lowest on day 4 does not establish that all athletes experience the same response. Substantial interindividual variation is expected (Chapman et al., 1998), and individualized monitoring (e.g., daily waking heart rate, SpO_2_, and subjective recovery markers) is recommended rather than universal prohibition of high-intensity exercise on any specific day.

### 4.6 Limitations

Several limitations directly affect the interpretation, causal attribution, and generalizability of the findings.

1. Small sample size: The study included only 14 participants, limiting statistical precision and increasing the influence of individual variation and outliers.
2. Absence of an *a priori* sample-size calculation: No formal power analysis was conducted prior to data collection; the sample size was based on practical feasibility.
3. Single-cohort design without a control group: Without a sea-level control group completing the same repeated testing schedule, altitude effects cannot be fully distinguished from repeated-testing effects, training fatigue, recovery patterns, or temporal variation.
4. Limited measurement time points: Measurements were conducted only at baseline and on days 1, 4, and 6. Therefore, the study cannot establish that the true nadir occurred specifically on day 4; the lowest value may have occurred at an unmeasured time between assessments.
5. Repeated maximal exercise testing: Four maximal or near-maximal exercise tests within a short period may have introduced cumulative fatigue, incomplete recovery, familiarization, pacing, or motivational effects.
6. Potential environmental confounding: Outdoor testing may have been influenced by temperature, humidity, wind, air quality, track conditions, or time of day.
7. Potential travel and behavioral confounding: Rapid relocation, rail travel, changes in sleep, diet, hydration, daily routines, and psychological stress may have influenced the observed responses.
8. Limited physiological measurements: Proposed autonomic, ventilatory, hematological, and metabolic mechanisms were not directly measured. Consequently, mechanistic interpretations remain speculative.
9. Restricted population: The findings are limited to a small sample of male national-level youth endurance athletes and cannot be generalized to female athletes, non-elite athletes, other sports, older adults, or individuals with different altitude experience.
10. Short observation period: The study evaluated only the first six days and therefore does not describe complete acclimatization or subsequent performance outcomes.

Each of these limitations reduces the strength of causal conclusions and underscores that the findings should be treated as hypothesis-generating rather than definitive.

## 5 Conclusion

Acute exposure to a sub-high altitude of 1,300 m induces a distinct, non-linear impairment in aerobic power and running economy in elite youth endurance athletes. The physiological nadir occurs consistently around Day 4, characterized by concurrent depressions in resting SpO_2_ elevated blood lactate accumulation, and elevated waking heart rates. By Day 6, early homeostatic re-stabilization and partial recovery of cardiorespiratory parameters become evident. Coaches may consider closer monitoring and temporary adjustment of training intensity during the early days following ascent, particularly around day 4, utilizing days 1–2 and day 6 as potential training micro-windows. These recommendations are preliminary and require validation in larger, controlled trials.

## Data Availability

The raw data supporting the conclusions of this article will be made available by the authors, without undue reservation.
